# AGE-BASED ENTITLEMENT: AN AGEIST PRACTICE OR A TOOL FOR COMBATTING AGEISM?

**DOI:** 10.1093/geroni/igad104.1987

**Published:** 2023-12-21

**Authors:** Tove Harnett, Håkan Jönson

**Affiliations:** Lund University, Lund, Skane Lan, Sweden; Lunds Universitet, Lund, Skane Lan, Sweden

## Abstract

Researchers in gerontology have addressed the way age-based arrangements may communicate stereotypical and devaluing images of older people, thereby linking high age to frailty and dependence. The present article considers proposed reforms to the Swedish eldercare system designed to guarantee people over 85 the right to move into a nursing home regardless of their needs. The purpose of the article is to investigate older people’s views on age-based entitlement in light of this proposal. What might the consequences of implementing the proposal be? Does it communicate devaluing images? Do the respondents consider it a case of ageism? The data consists of 11 peer group interviews with 34 older individuals. Bradshaw’s taxonomy of needs was used to code and analyze data. Four positions on the proposed guarantee were identified: care should be arranged: (1) according to needs, not age; (2) according to age as a proxy for needs; (3) according to age, as a right; and (4) according to age, to combat “fourth ageism”, meaning ageism directed towards frail older persons with care needs, i.e. persons in the fourth age . The notion that such a guarantee might constitute ageism was dismissed as irrelevant, while difficulties in getting access to care were presented as the real discrimination. It is theorized that some forms of ageism posited as theoretically relevant may not be experienced as such by older people themselves.

